# Different effects of propofol and dexmedetomidine sedation on electroencephalogram patterns: Wakefulness, moderate sedation, deep sedation and recovery

**DOI:** 10.1371/journal.pone.0199120

**Published:** 2018-06-19

**Authors:** Chunhua Xi, Shiyue Sun, Chuxiong Pan, Fang Ji, Xu Cui, Tianzuo Li

**Affiliations:** 1 Department of Anesthesiology, Beijing Tongren Hospital affiliated to Capital Medical University, Beijing, China; 2 Department of Psychology, Beijing Forestry University, Beijing, China; 3 Department of Anesthesiology, Beijing Shijitan Hospital affiliated to Capital Medical University, Beijing, China; Massachusetts General Hospital, UNITED STATES

## Abstract

Sedation induces changes in electroencephalography (EEG) dynamics. However, the distinct EEG dynamic characteristics at comparable sedation levels have not been well studied, resulting in potential interpretation errors in EEG monitoring during sedation. We aimed to analyze the EEG dynamics of dexmedetomidine and propofol at comparable sedation levels and to explore EEG changes with increased sedation levels for each agent. We measured the Bispectral Index (BIS) and 20-channel EEG under dexmedetomidine and propofol sedation from wakefulness, moderate sedation, and deep sedation to recovery in healthy volunteers (n = 10) in a randomized, 2-day, crossover study. Observer’s Assessment of Alertness and Sedation (OAA/S) score was used to assess sedation levels. Despite similar changes in increased delta oscillations, multiple differences in the EEG spatiotemporal dynamics were observed between these two agents. During moderate sedation, both dexmedetomidine and propofol induced increased spindle power; however, dexmedetomidine decreased the global alpha/beta/gamma power, whereas propofol decreased the alpha power in the occipital area and increased the global spindle/beta/gamma power. During deep sedation, dexmedetomidine was associated with increased fronto-central spindle power and decreased global alpha/beta/gamma power, but propofol was associated with increased theta/alpha/spindle/beta power, which was maximized in the frontal area. The transition of topographic alpha/spindle/beta power distribution from moderate sedation to deep sedation completely differed between these two agents. Our study demonstrated that there was a distinct hierarchy of EEG changes with increased sedation depths by propofol and dexmedetomidine. Differences in EEG dynamics at the same sedation level might account for differences in the BIS value and reflect the different sedation mechanisms. EEG-based clinical sedation monitoring should consider the effect of drug types on EEG dynamics.

## Introduction

Sedation is a state of altered consciousness induced by different kinds of anesthetics. Sedation can provide a comfortable experience for the patient and a better operating condition for the clinician during unpleasant diagnostic or therapeutic procedures, and it is now widely performed in operating rooms and non-operating sites[1–3]. Sedation ranges from minor antianxiety, amnesia, and hypnosis to unconsciousness according to the clinical requirements. Conscious sedation is a state that allows patients to tolerate unpleasant procedures when they are awake and collaborative, while deep sedation provides a condition of no response to auditory stimuli or noxious stimulation[4, 5]. The graded fashion of conscious experience might reflect hierarchical brain dynamics. Electroencephalogram (EEG) measures cortical brain activity. However, more studies have focused on the transition from wakefulness to unconsciousness, and the different levels of sedation are not well characterized by EEG[6, 7].

Sedatives might act at different molecular targets and neural circuits to produce distinct EEG traces[8]. The common sedatives that alter arousal states include gamma-amino butyric acid type A (GABA_A_) receptor agonists, opioid receptor agonists, N-methyl D-aspartate receptor (NMDA) antagonists and α2 receptor agonists. Previous studies have demonstrated a drug-dependent EEG trace during sedation. Propofol, an agonist of GABA_A_ receptors, induces loss of consciousness (LOC) and is characterized by an abrupt anteriorization of alpha rhythms[6,9–11]. This distinct EEG variation is also observed during halothane, isoflurane, sevoflurane, and desflurane anesthesia[12–14]. Ketamine, an NMDA antagonist, results in “gamma bursts” and markedly increases the theta power across the cortex[7, 15]. Dexmedetomidine is a highly selective α2-adrenergic receptor agonist. Dexmedetomidine-induced sedation resembles non-rapid eye movement (NREM) sleep with a characteristic spindle wave (12–16 Hz) in the frontal area[16, 17]. EEG dynamics also change with the agent dose and sedation level. Moderate propofol sedation increases EEG oscillations in the spindle range (12–15 Hz) and beta range (13–25 Hz) in frontal areas, and deep sedation is associated with delta oscillations across the cortex and alpha anteriorization[11]. Ketamine at a dose of 0.5 mg/kg is characterized by reduced posterior alpha power, and 1.5 mg/kg is associated with increased delta, theta and gamma power across the cortex[15]. However, whether the above EEG dynamics are comparable at the same clinical sedation level for different sedatives and whether EEG parameters are suitable for monitoring the depth of sedation remain unknown.

Describing EEG dynamics during a comparable sedation level for different sedatives might help explain the possibility of EEG monitoring during sedation and, to some extent, explore sedation mechanisms. Propofol and dexmedetomidine are two popular sedatives. Although the two agents can induce similar sedation levels by clinical evaluation, there is a significant difference in Bispectral Index (BIS) monitoring at the same sedation level[18]. This study aimed to determine the effect of propofol and dexmedetomidine on EEG spectral power parameters and EEG topography within specific frequency bands during different depths of sedation. We hypothesized that propofol and dexmedetomidine induce different brain dynamics at comparable sedation levels and that a distinct hierarchy of EEG changes exists with the increased sedation level for each agent. To explore these hypotheses, we measured and analyzed the EEG patterns of 10 healthy volunteers who received both propofol and dexmedetomidine sedation in a crossover study.

## Materials and methods

### Subjects and EEG data collection

The Ethics Committee of Beijing Tongren Hospital affiliated to Capital Medical University provided ethical approval for this study on 19, October 2015 (Chairperson Prof. Ningli Wang, protocol number TRECKY2015-021). The study was registered with the Chinese Clinical Trial Registry (ChiCTR-OON-16008369). Ten healthy male volunteers (ASA physical status I, right-handed, non-smoking, normal weight) aged 23 to 30 were enrolled in this study after public advertisements. Each volunteer was informed of the consent policies, and written consent was obtained. All subjects underwent a physical examination, including an interview, physical status assessment, laboratory testing and a 12-lead ECG examination. Subjects were not allowed to use alcohol or any medication 48 hours before the study, and all fasted for at least 8 hours. All experimental procedures were scheduled from 9 am to 12 pm in a standard operating room to avoid the influence of circadian rhythm on sedation. We performed a randomized, 2-day crossover study design for propofol/dexmedetomidine sedation, with one sedation procedure on the first day and the other sedation procedure two weeks later. Volunteers were assigned to receive either propofol or dexmedetomidine according to a computer-generated randomization during the first sedation procedure. Oxygen supplement (2 l/min) was provided via a mask. Sedation monitoring included the BIS Index (BIS VISTA^TM^ monitor, software 2.00, Aspect Medical Systems, Newton, MA), noninvasive arterial pressure (NIBP), a three-lead electrocardiogram, pulse oximetry, and respiratory rate.

After resting for 5 min, the volunteers were instructed to close their eyes and listen to a 2-min auditory stimulus. For dexmedetomidine sedation, dexmedetomidine (dexmedetomidine hydrochloride solution, 100 μg/ml, Hengrui, Jiangsu, China) diluted to 4 μg/ml was administered intravenously with a loading dose of 1 μg/kg over 10 min and a maintenance dose of 0.4–1 μg/kg/h. Propofol sedation was given via target-controlled infusion (TCI) with the pharmacokinetic parameters obtained from Schnider[19]. The target effect-site concentration of propofol was set beginning at 1 μg/ml and was then increased by 0.3 μg/ml per step. The 5-point Observer's Assessment of Alertness/Sedation Scale (OAA/S) was used every 3 min to determine the sedation level in each case and to help titrate the infusion rate[18]. Wakefulness was defined as volunteers without any drug administration, a moderate sedation state was defined as OAA/S = 3 (responds only after name is spoken loudly and/or repeatedly), deep sedation was defined as OAA/S = 1 (does not respond to mild prodding or shaking), and recovery was defined as OAA/S = 5 (responds readily to name spoken in a normal tone)[18].

**Fig 1:**
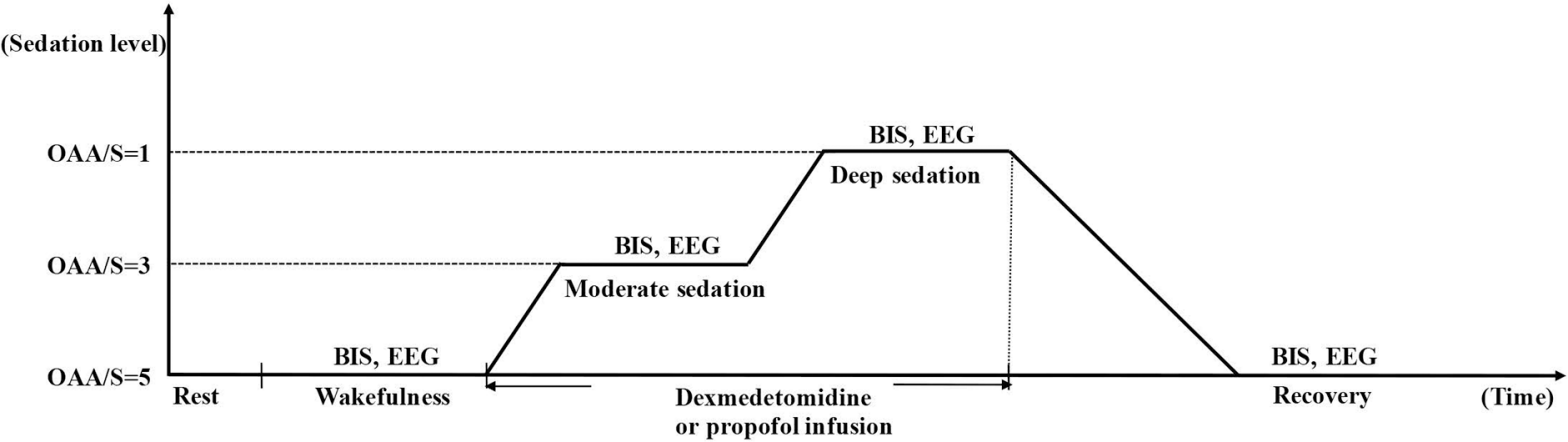
Experimental paradigm. Volunteers underwent Bispectral monitoring and EEG recording during each of the four experimental sessions: wakefulness, moderate sedation (OAA/S = 3), deep sedation (OAA/S = 1), and recovery (OAA/S = 5). Dexmedetomidine or propofol was administered intravenously and titrated to achieve the required sedation level.

The CogniTrace 20-channels system (A.N.T., Netherlands) was applied to record the continuous EEG and play the auditory stimuli. The EEG signals from 20 sites were based on the international 10–20 system. All EEG electrodes were referred to the mastoids (M1 + M2 average). The impedance of each electrode was kept below 5 kΩ. The auditory stimuli (2000 Hz, 75 dB, with intervals of one s) were repeatedly displayed and applied via earphones at wakefulness, moderate sedation, deep sedation and recovery. The study design is provided in Fig 1.

### EEG spectral analysis and topographic mapping

After data collection, the EEG was analyzed offline with EEG lab 14.0 software as Zhang et al. described[20, 21]. Briefly, all EEG data were filtered offline with a 0.5–100 Hz bandpass filter for analysis. Filtered EEG data were then visually inspected for artifacts. The artifact rejection methods consisted of the exclusion of epochs with a large amplitude (over ±100 μV), DC bias, blinks, and slow eye movement. Bad electrodes were substituted with the extrapolated virtue values from the neighboring channels. After artifact rejection, each set of EEG data was subjected to a 2-s epoch, and each epoch was processed using fast Fourier transformation (FFT) analysis to obtain the absolute power at each electrode in the following six bands: delta (0.5–4 Hz), theta (4–8 Hz), alpha (8–12 Hz), spindle (12–15 Hz), beta (15–25 Hz) and gamma (25–40 Hz). In each 2-min period, EEG was analyzed in 2-s epochs, resulting in 60 epochs. On average, approximately 50–58 valid epochs in each study condition were subjected to further analyses. Spectral power was estimated by FFT, with window lengths of T = 1 s with a 0-s overlap and a spectral resolution of 0.25 Hz. The topographic power distribution was calculated with the EEGLab toolbox[22].

### Statistical analysis

Sedation parameters are expressed as the mean ± SD. Statistical analyses of the sedation parameters were performed using GraphPad Prism 6.01 (GraphPad Prism Software Inc., La Jolla, California, USA). The sedation times of the two agents were compared with unpaired t-tests. Two-way repeated measures analysis of variance (RM-ANOVA) and Bonferroni's multiple comparisons test were used to assess the variations in BIS values in the drug regimes (dexmedetomidine and propofol) and the sedation level (wakefulness, moderate sedation, deep sedation and recovery state). Low power spectral density, 10*log_10_ (μV^2^/Hz), was used to describe the absolute spectral power. For each participant, the power spectrum density and statistical analyses were conducted with the Matlab-based EEGLab toolbox[22]. According to previous literature and the current topography results, sedation states induce peak spectrum activity at the frontal midline electrodes. Therefore, the Fz electrode spectra were analyzed with two-way RM-ANOVA followed by planned pairwise comparisons between moderate sedation and baseline, deep sedation and baseline, recovery state and baseline as well as between deep and moderate sedation conditions for both dexmedetimidine and propofol. To explore the long-range coordination of neural activity, all 20 electrodes were included in the topographic analysis. The signals were divided into six frequency bands: delta, theta, alpha, spindle, beta and gamma. For each band, ANOVAs and planned comparisons similar to those used for the Fz spectral analyses were performed to explore the topographical differences. All statistical significances of the Fz and topographical analyses were calculated using a nonparametric bootstrap approach with a 10000 resampling size at alpha = 0.05. Multiple comparisons were corrected by the false discovery rate (FDR) procedure, a commonly used method for high-dimension data analysis.

## Results

### Participant characteristics and sedation parameters

Ten right-handed male volunteers took part in this study, but two could not reach the deep sedation state during the dexmedetomidine infusion. We did not increase the dexmedetomidine dosage because of safety issues. Therefore, data from the remaining 8 volunteers were included in the final analysis. The times from wakefulness to moderate sedation, from moderate sedation to deep sedation and from deep sedation to recovery during dexmedetomidine sedation were 10.8 ± 2.5 min, 15.4 ± 6.8 min and 52.6 ± 14.6 min, respectively. The times from wakefulness to moderate sedation, from moderate sedation to deep sedation and from deep sedation to recovery during propofol sedation were 10.4 ± 3.5 min, 13.5 ± 5.3 min and 22.8.6 ± 5.4 min, respectively. There were no significant differences in the time from wakefulness to moderate sedation or the time from moderate sedation to deep sedation between dexmedetomidine and propofol sedation (*P* = 0.81 and *P* = 0.55, respectively); however, the time from deep sedation to recovery during dexmedetomidine sedation was significantly longer than that of propofol (*P*<0.0.001).

**Fig 2:**
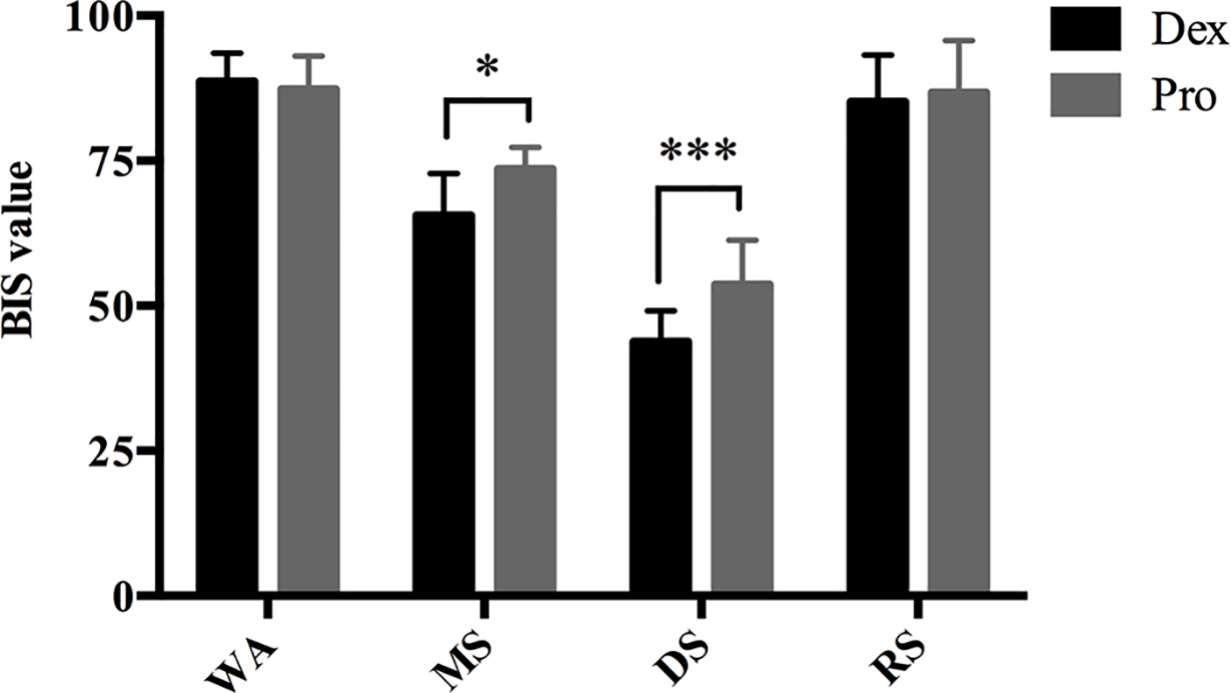
Bispectral (BIS) value during the dexmedetomidine/propofol sedation procedure. Data are shown as the mean ± standard deviation. *P< 0.05, ***P <0.001. WA, wakefulness; MS, moderate sedation; DS, deep sedation; RS, recovery state (RS).

The BIS values at wakefulness, moderate sedation, deep sedation and recovery during dexmedemidine sedation were 88.8 ± 5.0, 65.6 ± 7.1, 43.8 ± 5.3 and 85.1 ± 8.1, respectively. The BIS values at wakefulness, moderate sedation, deep sedation and recovery during propofol sedation were 87.2± 5.8, 73.6 ± 3.7, 53.6 ± 7.6 and 86.7 ± 9.0 respectively. The BIS values at moderate and deep sedation by dexmedetomide were significantly lower than those by propofol (sedation effect: *P*<0.001, drug effect: *P* = 0.019, sedation*drug interaction: *P* = 0.016, Fig 2).

### Frontal spectral power changes from wakefulness, moderate sedation, and deep sedation to recovery during dexmedetomidine/propofol sedation

**Fig 3:**
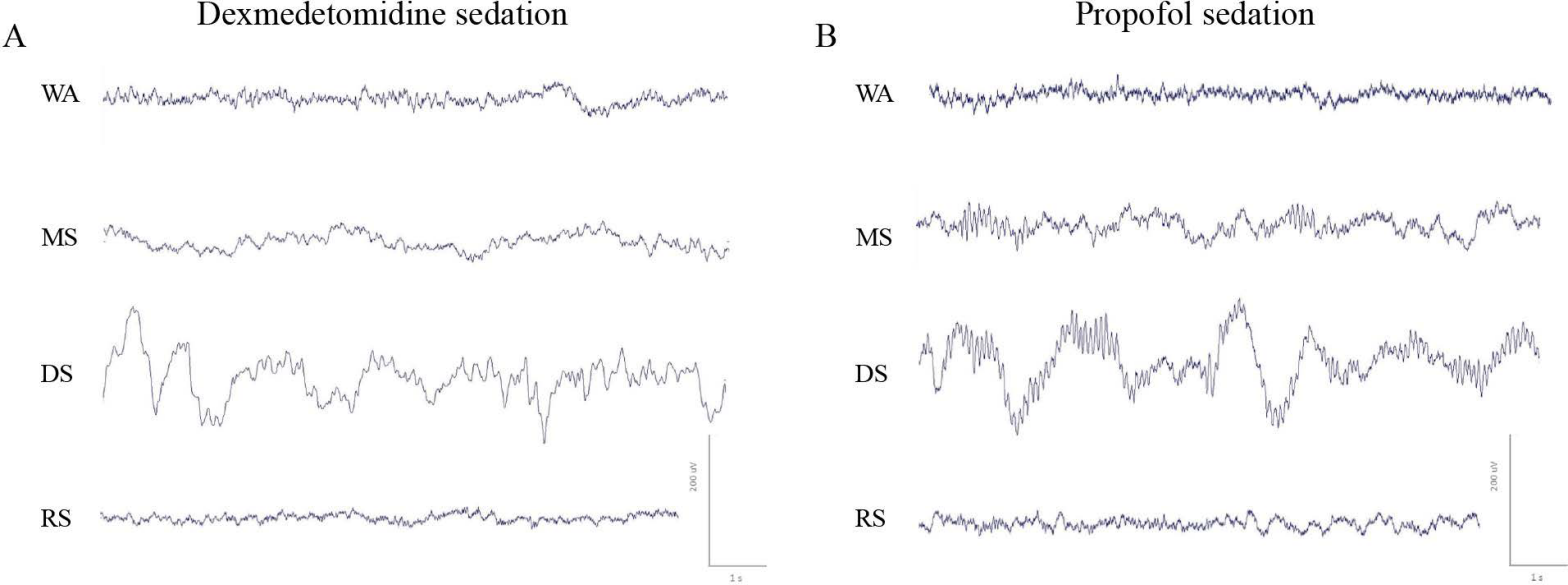
Changes in a raw EEG trace of dexmedetomidine sedation and propofol sedation from channel Fz. WA, wakefulness; MS, moderate sedation; DS, deep sedation; RS, recovery state (RS). During MS and DS, the EEG differences between the two agents are obvious.

**Fig 4:**
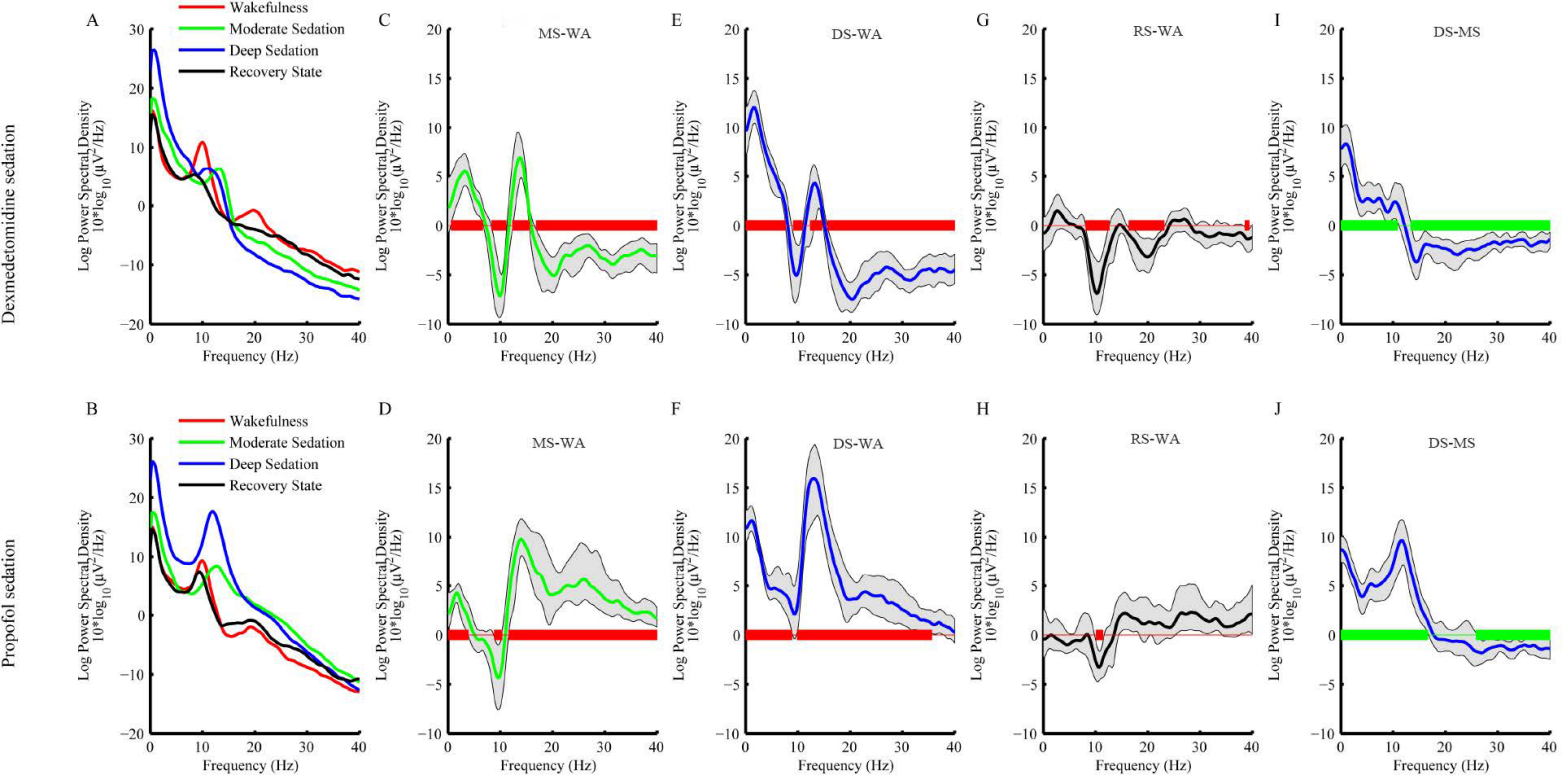
Spectral analysis of EEG from channel Fz for both dexmedetomidine sedation and propofol sedation. (A-B) Mean power spectra of WA (red), MS (green), DS (blue) and RS (black) for the two agents. (C-D) The green line represents the bootstrapped mean spectra of the difference between MS and WA for the two agents, and the gray space represents the bootstrapped 95% confidence interval bounds for the difference. (E-F) The blue line represents the bootstrapped mean spectra of the difference between DS and WA for the two agents, and the gray space represents the bootstrapped 95% confidence interval bounds for the difference. (G-H) The black line represents the bootstrapped mean spectra of the difference between RS and WA for the two agents, and the gray space represents the bootstrapped 95% confidence interval bounds for the difference. (I-J) The blue line represents the bootstrapped mean spectra of the difference between DS and MS for the two agents, and the gray space represents the bootstrapped 95% confidence interval bounds for the difference. The horizontal solid red lines represent the frequency ranges at which significant differences exist between each sedation state and WA, and the solid green lines represent the frequency ranges at which significant differences exist between DS and MS. WA, wakefulness; MS, moderate sedation; DS, deep sedation; RS, recovery state (RS).

One volunteer’s raw EEG of Fz and spectral analysis of Fz during the sedation procedures are presented (Fig 3 and Fig 4). We observed changes in the spectral analysis that were induced by dexmedetomidine and propofol from wakefulness to recovery, and the changes tended to differ between these two agents. Statistical analysis showed significant differences in the ranges 0–8 Hz, 9.25–16.25 Hz, and 31.5–40 Hz for the main sedation effect, 10.5–30 Hz for the main drug effect, and 10–40 Hz for the interaction effect. Multiple comparisons showed that compared with wakefulness, the dexmedetomidine-induced spectral power of moderate sedation was larger at 0.75–6.5 Hz and 12.5–15.5 Hz and smaller at 8.5–11 Hz and 17.5–40 Hz, whereas the propofol-induced spectral power of moderate sedation was larger at 0.25–3.75 Hz and 11.75–40 Hz and smaller at 9–10.25 Hz. Additionally, the dexmedetomidine-induced spectral power of deep sedation was larger at 0.25–7.5 Hz and 12.5–14.5 Hz and smaller at 9.25–10.75 Hz and15.75–40 Hz, while the propofol-induced spectral power of deep sedation was larger at 0.25–8.75 Hz and 10–35.5 Hz. Further, the dexmedetomidine-induced spectral power of recovery was smaller at 8.25–12. 5 Hz, 16.5–23 Hz and 38.75–39.25 Hz, whereas the propofol-induced spectral power of recovery was smaller at 10–11.25 Hz. Compared with moderate sedation, deep dexmedetomidine sedation was associated with increased spectral power in the range of 0.25–10.75 Hz and decreased spectral power in the range of 13.5–40 Hz, while the deep propofol sedation was associated with increased spectral power in the range of 0.25–16.5 Hz and decreased spectral power in the range of 26–40 Hz. Thus, for these two agents, the spectral powers of the alpha, spindle, beta and gamma bands changed differently from wakefulness to recovery. Moreover, the spectral power changes from moderate sedation to deep sedation were different in the alpha, spindle, and beta bands.

### Topographic changes in EEG power from wakefulness, moderate sedation, and deep sedation to recovery during dexmedetomidine/propofol sedation

**Fig 5:**
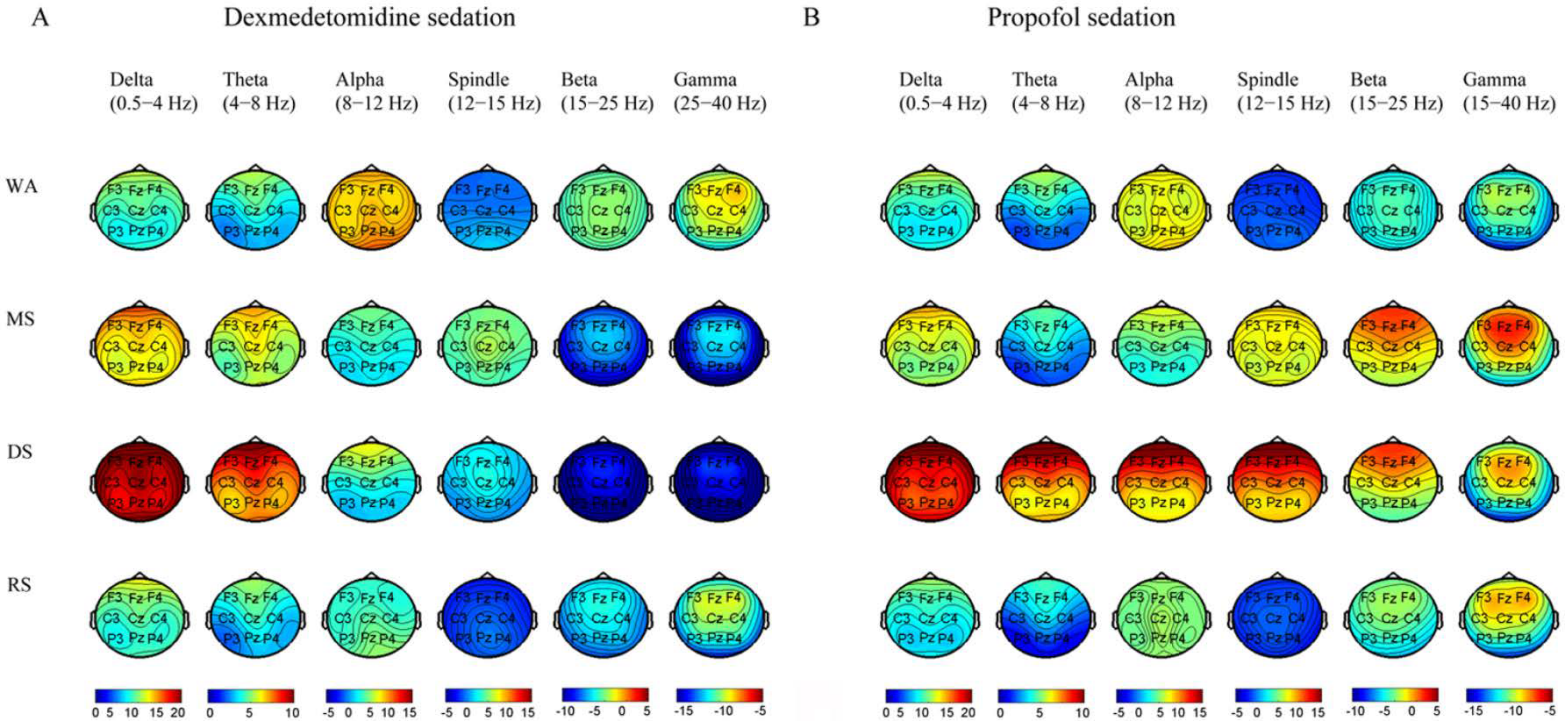
Topographic EEG maps of the spectral power of each frequency band for both dexmedetomidine sedation and propofol sedation. Delta (0.5–4 Hz), theta (4–8 Hz), alpha (8–12 Hz), spindle (12–15 Hz), beta (15–25 Hz) and gamma (25–40 Hz). The maps show the total power (10*log_10_ (μV^2^/Hz)). WA, wakefulness; MS, moderate sedation; DS, deep sedation; RS, recovery state (RS).

Both dexmedetomidine- and propofol-induced topographic changes in the EEG power of different bands during the sedation procedure are represented in Fig 5. As typically observed, both dexmedetomidine and propofol were correlated with increased global delta band power, but changes in other bands were topographically distinct, especially in the alpha, spindle, beta and gamma ranges. Statistical analysis showed significant differences in all electrodes of the six bands for the main sedation effect, in all electrodes of the spindle and beta bands for the main drug effect and in all electrodes of the alpha, spindle, beta and gamma bands for the interaction effect.

**Fig 6:**
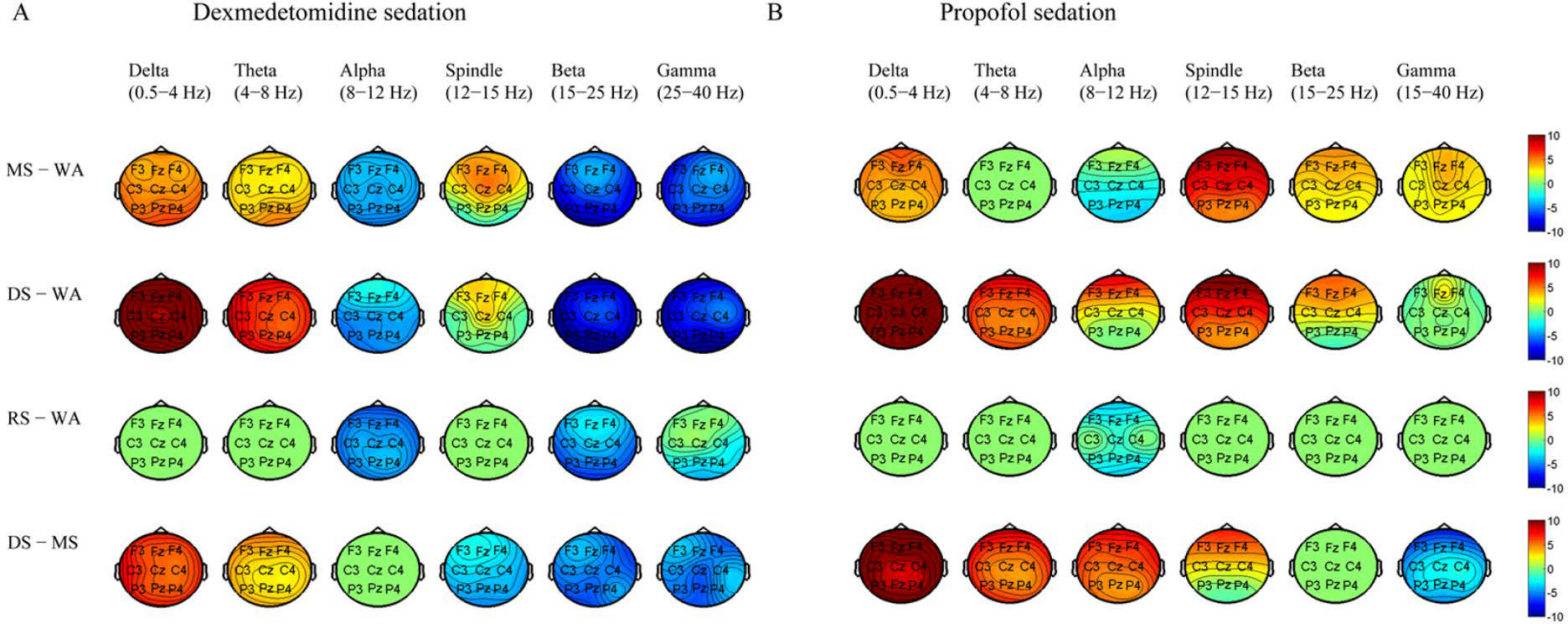
Topographic EEG changes in the spectral power of each frequency band for both dexmedetomidine sedation and propofol sedation. Delta (0.5–4 Hz), theta (4–8 Hz), alpha (8–12 Hz), spindle (12–15 Hz), beta (15–25 Hz) and gamma (25–40 Hz). The maps show the differences between two states (MS-WA, DS-WA, RS-WA and DS-MS) for each frequency band of interest as a t-statistic. WA, wakefulness; MS, moderate sedation; DS, deep sedation; RS, recovery state (RS).

To identify the spectral changes, differences between the two states are displayed as changes in power with its associated t-statistic in Fig 6. Moderate dexmedetomidine sedation was associated with a reduction in global alpha, beta, gamma band power and an increase in global delta, theta power and spindle power at the frontal-vertex sites; by contrast, moderate propofol sedation was associated with a reduction in occipital alpha power and increases in delta, spindle, beta and gamma power at the global cortex. During deep dexmedetomidine sedation, global delta and theta power continued to increase, and global beta and gamma power continued to decrease, whereas deep propofol sedation was associated with the sustained increase in global delta power and increased theta/alpha/spindle/beta power maximally in the frontal area. The alpha/beta oscillation power was persistently lessened across the entire cortex at the recovery state from dexmedetomidine, while there was a minor reduction in alpha power at the frontal and occipital areas with the recovery state from propofol. Compared with moderate sedation, dexmedetomidine increased the delta and theta power but decreased the spindle, beta and gamma power across the cortex region during deep sedation, whereas propofol increased the global delta, theta, alpha band power, decreased the global gamma power, and strengthened the spindle power in the fronto-central area. The above data suggest that although the topographic changes in delta power were similar to those from wakefulness to recovery for the two agents, the powers of the other bands differed. The transition of topographic alpha/spindle/beta power distribution from moderate sedation to deep sedation between these two agents is completely different.

## Discussion

In this study, we visualized gradual changes in EEG dynamics from wakefulness, moderate sedation, and deep sedation to recovery during propofol/dexmedetomidine sedation. We demonstrated that there was a distinct hierarchy of EEG changes with increased sedation levels for these two agents. Despite a similar change in sustained increase in delta oscillation from wakefulness to deep sedation, multiple differences in the EEG spatiotemporal dynamics were observed between the two agents. 1) During moderate sedation, both dexmedetomidine and propofol induced increased spindle power; however, dexmedetomidine increased theta power and decreased alpha/beta/gamma power across the whole cortex, whereas propofol decreased alpha power in the occipital area and increased global beta/gamma power. 2) During deep sedation, dexmedetomidine was associated with increased global theta power and fronto-central spindle power and decreased alpha/beta/gamma power across the whole cortex, but propofol was associated with increased theta/alpha/spindle/beta power, which was maximized in the frontal area. 3) The transition of topographic alpha/spindle/beta power distribution from moderate sedation to deep sedation completely differed between dexmedetomidine and propofol.

### Different frontal spectral powers and BIS differences at the same sedation levels

Like natural sleep, sedation is a state of decreased arousal. The clinical manifestation ranges from drowsiness to unconsciousness. Sleep is characterized into rapid eye movement (REM) and three stages of NREM sleep by the distinct EEG patterns. However, the application of EEG-based monitoring to measure the depth of sedation is controversial.

The BIS value is calculated from three components: spectral analysis, bispectral analysis and temporal analysis[23]. The frequency domain-based relative β ratio is measured by the spectral analysis. The β ratio is defined as the logarithm of the power ratios in two empirically derived frequency bands, log (P_30-47Hz_)/(P_11-20Hz_), and is a major parameter for the BIS calculation of the sedation level[24]. In our study, a substantial difference in the frontal spectral power was observed in the 10–40 Hz range during the sedation procedures between dexmedetomidine and propofol. The frontal beta (15–25 Hz) and gamma (25–40 Hz) powers were decreased by dexmedetomidine and increased by propofol. This result sufficiently explains why the BIS value of dexmedetomidine was lower than that of propofol at comparable sedation levels in our study and in previous report[18, 25]. Additionally, it explains the decreased BIS value when dexmedetomidine was added to propofol anesthesia as well as the reduction in propofol required during BIS-guided closed-loop anesthesia when dexmedetomidine was added[26–28].

### Different spatiotemporal alpha/spindle/beta/gamma oscillations during dexmedetomidine/propofol sedation

As the targets of propofol, GABA_A_ receptors are widely distributed in the cortex, the thalamus and the preoptic area (POA) of the hypothalamus. The mechanisms underlying propofol-mediated sedation include enhancement of GABA_A_ inhibition from inhibitory interneurons to excitatory pyramidal neurons, from the thalamus to the cortex and from the POA to the arousal centers, including the midbrain, the pons, and the hypothalamus[29–31]. Dexmedetomidine selectively acts on the α_2_- receptors of the locus coeruleus (LC) projecting to the POA, which activates the inhibitory outputs to the arousal centers and results in a sedative state[30, 32]. Recently, the thalamocortical system was shown to contribute to both propofol and dexmedetomidine-induced altered arousal[10, 33, 34]. However, the effects of propofol and dexmedetomidine on the thalamocortical system have never been compared.

Alpha rhythms (8–12 Hz) are spontaneous EEG oscillatory activities over the occipital-parietal cortex of awake humans in a relaxed state with their eyes closed, and these rhythms change with the arousal state and cognitive activities. Spindles (12–15 Hz) are one of the most dominant EEG oscillations during NREM stage 2 sleep, and they have a lower density in deeper slow-wave sleep (SWS). Both the alpha power and the spindle power change in all parts of the cortex from wakefulness to different stages of sleep[35–38]. The origin of spindle waves shares a similar mechanism with alpha waves, as the GABAergic reticular nucleus in the thalamus is the pacemaker of this spindle rhythm, and the thalamocortical neurons and cortical neurons potentiate the genesis of spindle waves[39–42]. Propofol-induced LOC is characterized by an abrupt anteriorization of alpha rhythms[6,9–11]. Using a Hodgkin–Huxley-based model, Vijayan and Kopell suggested that alpha rhythms arise via a specialized class of thalamocortical cells (TCs), so-called high-threshold thalamocortical neurons (HTCs)[43]. Based on the above model, a computational model suggested the following: 1) Propofol attenuates the occipital alpha power by silencing HTCs via reducing the hyperpolarization-activated current I_*h*_. 2) Propofol potentiates the GABA_A_ synaptic current and decay time from interneurons onto pyramidal neurons cells and from reticular neurons (REs) onto TCs, thus bringing alpha activity to the cortex, creating alpha oscillations between REs and TCs, and enhancing the cortical inputs to the thalamus. These alternations result in a reciprocal excitation between the cortex and thalamus and form a strengthened cortical-thalamo-cortical loop alpha oscillation[10, 44]. In our study, we confirmed the hierarchical changes in the propofol-induced reduction of occipital alpha oscillations during moderate sedation and propofol-induced alpha anteriorization during LOC. The topographic representation of alpha activity changes by dexmedetomidine is similar to that of sleep, with attenuation of alpha power across the frontal-posterior cortex[35–37]. The effect of dexmedetomidine on the HTCs has not been described. The neurotransmitter norepinephrine enhances the hyperpolarization-activated current I_*h*_ in thalamocortical neurons in vitro[45]. Dexmedetomidine decreases norepinephrine release from the LC, which might reduce the hyperpolarization-activated current I_*h*_ and lead to attenuated alpha oscillation. Whether reducing the hyperpolarization-activated current I_*h*_ is involved in the mechanisms of dexmedetomidine-induced sedation needThe lack of alpha anteriorization might be possibly due to the short GABA_A_ potentiation by dexmedetomidine.

Both dexmedetomidine and propofol sedation result in the appearance of spindle activity[17, 46]. In our study, dexmedetomidine–induced spindle oscillations increased dramatically during moderate sedation and attenuated during deep sedation, which shared a similar tendency with spindles across varying depths of NREM[38]. For propofol, increased spindle activity from frontal to posterior areas was observed during moderate sedation, and deep sedation dramatically increased the spindle activity in the frontal regions. These results are consistent with a previous report that described propofol-induced LOC being accompanied by increased spindle oscillations in the frontal area with/without auditory stimuli[11]. Although the increased spindle oscillations share a similar topographic pattern as alpha oscillations during deep sedation, their differential characteristics during wakefulness and moderate sedation indicate the impossibility of overlapping analysis. The distinct changes in spindle oscillation of the two agents also suggest the different thalamocortical sedation mechanisms. Recently, the slow spindles (centered at approximately 12 Hz) and fast spindles (centered at approximately 14 Hz) were found to differ in their frequency, topographic distribution and slow oscillation cycle points[38, 47–49]. The slow fast spindles are generated by different mechanisms and may reflect different physiological processes[38, 48]. One possible explanation may be based on the anatomical heterogeneity because spindles from the core and matrix thalamus system supposedly differ in their cortical projections[50]. The loss of thalamocortical functional connectivity presumably induces both propofol and dexmedetomidine-induced unconsciousness, and propofol confers differential changes in functional connectivity of the different parts of the thalamocortical systems[29, 33]. However, in our study, we could not deduce whether the differential effect of propofol and dexmedetomidine on spindle oscillations was attributed to their distinct effects on the fast/slow spindles or on the different anatomical locations of the thalamocortical systems. Further study combining electrophysiology and functional neuroimaging might explore their different thalamocortical mechanisms. The different spatiotemporal dynamics of alpha and spindle oscillations also suggest that propofol and dexmedetomidine act differently in the thalamocortical system.

The beta/gamma rhythms have been associated with cortical activities and higher levels of cognitive activities, such as sensory gating, attention, perception and motor control. In our study, propofol and dexmedetomidine had different effects on beta/gamma rhythms during sedation, and dexmedetomidine persistently decreased global beta/gamma oscillations during sedation and recovery. The decreased beta/gamma oscillations accounted for the lower BIS value of dexmedetomidine; however, elucidating these central mechanisms was beyond the scope of our study.

### Clinical implications

The application of EEG-based monitoring to measure the depth of sedation is controversial.

The BIS monitor displays a real-time EEG trace acquired from a frontotemporal montage. Good correlation exists between the BIS value and the dexmedetomidine/propofol-induced sedation level[18, 51]. However, the BIS value does not change with increased sedation depth with nitrous oxide or ketamine[52–55]. Moreover, when ketamine is used as an adjuvant of other anesthetics, such as propofol or sevoflurane, it reportedly has no effect or even increases the BIS value[54, 56–58]. The results of our study indicate that the different thalamocortical mechanisms might play an important role in propofol and dexmedetomidine-induced different EEG frontal spectral power, which results in their distinct BIS values at the same sedation level. We suggest that when BIS is used to monitor the depth of sedation or anesthesia, more consideration should be paid to the types of sedatives/anesthetics and their specific targets and neural circuits in the central nervous system. Furthermore, using the BIS value to compare the depths of sedation/anesthesia for different drugs might not be appropriate.

### Limitations

In this study, we performed spectral power analysis and created topographic maps to demonstrate gradual brain dynamic changes from wakefulness, moderate sedation, and deep sedation to recovery. We did not apply global or local coherence analysis; thus, the degree of correlation between two cortical regions could not be reflected. Additionally, due to the limitation of EEG recording, we could not directly determine the different effects of these two agents on the thalamus or thalamocortical system, and further study with the combination of functional neuroimaging might refine our results. Furthermore, although the thalamo-cortical network should be involved in propofol/dexmedetomidine-induced sedation, the cellular and neurochemical basis has been studied less. Determining whether reducing the hyperpolarization-activated current I_*h*_ is involved in the mechanisms underlying dexmedetomidine-induced sedation is a current research focus. Applying the different neuromodulators in microdialysis and the local field potential recording of animals might help further explore the sedation mechanisms at cellular and molecular levels.

## Conclusion

In this study, we demonstrated the existence of a distinct hierarchy of EEG changes with the increased sedation levels induced by propofol and dexmedetomidine. Although both agents induced similar increases in delta oscillations, multiple differences in alpha/spindle and beta/gamma oscillations at comparable sedation levels were observed. Based on our results and discussion, these differences might account for the difference in BIS values at the same sedation level and reflect the different sedation mechanisms. EEG-based clinical sedation monitoring should consider the effect of drug type on EEG dynamics.

## References

[1] Lamperti M. Adult procedural sedation: an update. Curr Opin Anaesthesiol. 2015;28(6):662–7. Epub 2015/09/12. doi: 10.1097/ACO.0000000000000244 . 26356290 10.1097/ACO.0000000000000244

[2] Mahmoud M, Mason KP. A forecast of relevant pediatric sedation trends. Curr Opin Anaesthesiol. 2016;29 Suppl 1:S56–67. Epub 2016/03/02. doi: 10.1097/aco.0000000000000321 . 26926335 10.1097/ACO.0000000000000321

[3] Sheahan CG, Mathews DM. Monitoring and delivery of sedation. Br J Anaesth. 2014;113 Suppl 2:ii37–47. Epub 2014/12/17. doi: 10.1093/bja/aeu378 . 25498581 10.1093/bja/aeu378

[4] Lerch C, Park GR. Sedation and analgesia. Br Med Bull. 1999;55(1):76–95. . 10695080 10.1258/0007142991902303

[5] Becker DE. Pharmacodynamic considerations for moderate and deep sedation. Anesthesia progress. 2012;59(1):28–42. Epub 2012/03/21. doi: 10.2344/0003-3006-59.1.28 ; PubMed Central PMCID: PMC3309299. 22428972 10.2344/0003-3006-59.1.28 PMC3309299

[6] Purdon PL, Pierce ET, Mukamel EA, Prerau MJ, Walsh JL, Wong KF, et al Electroencephalogram signatures of loss and recovery of consciousness from propofol. Proc Natl Acad Sci U S A. 2013;110(12):E1142–51. doi: 10.1073/pnas.1221180110 . 23487781 10.1073/pnas.1221180110 PMC3607036

[7] Akeju O, Song AH, Hamilos AE, Pavone KJ, Flores FJ, Brown EN, et al Electroencephalogram signatures of ketamine anesthesia-induced unconsciousness. Clin Neurophysiol. 2016;127(6):2414–22. doi: 10.1016/j.clinph.2016.03.005 . 27178861 10.1016/j.clinph.2016.03.005 PMC4871620

[8] Purdon PL, Sampson A, Pavone KJ, Brown EN. Clinical Electroencephalography for Anesthesiologists: Part I: Background and Basic Signatures. Anesthesiology. 2015;123(4):937–60. doi: 10.1097/ALN.0000000000000841 . 26275092 10.1097/ALN.0000000000000841 PMC4573341

[9] Ching S, Cimenser A, Purdon PL, Brown EN, Kopell NJ. Thalamocortical model for a propofol-induced alpha-rhythm associated with loss of consciousness. Proc Natl Acad Sci U S A. 2010;107(52):22665–70. doi: 10.1073/pnas.1017069108 . 21149695 10.1073/pnas.1017069108 PMC3012501

[10] Vijayan S, Ching S, Purdon PL, Brown EN, Kopell NJ. Thalamocortical mechanisms for the anteriorization of alpha rhythms during propofol-induced unconsciousness. J Neurosci. 2013;33(27):11070–5. doi: 10.1523/JNEUROSCI.5670-12.2013 . 23825412 10.1523/JNEUROSCI.5670-12.2013 PMC3718379

[11] Murphy M, Bruno MA, Riedner BA, Boveroux P, Noirhomme Q, Landsness EC, et al Propofol anesthesia and sleep: a high-density EEG study. Sleep. 2011;34(3):283–91A. Epub 2011/03/02. ; PubMed Central PMCID: PMCPMC3041704. 21358845 10.1093/sleep/34.3.283 PMC3041704

[12] Vijayan S, Ching S, Purdon PL, Brown EN, Kopell NJ. Biophysical Modeling of Alpha Rhythms During Halothane-Induced Unconsciousness. Int IEEE EMBS Conf Neural Eng. 2013:1104–7. doi: 10.1109/NER.2013.6696130 . 25284633 10.1109/NER.2013.6696130 PMC4180520

[13] Akeju O, Hamilos AE, Song AH, Pavone KJ, Purdon PL, Brown EN. GABAA circuit mechanisms are associated with ether anesthesia-induced unconsciousness. Clin Neurophysiol. 2016;127(6):2472–81. doi: 10.1016/j.clinph.2016.02.012 . 27178867 10.1016/j.clinph.2016.02.012 PMC4869993

[14] Akeju O, Westover MB, Pavone KJ, Sampson AL, Hartnack KE, Brown EN, et al Effects of sevoflurane and propofol on frontal electroencephalogram power and coherence. Anesthesiology. 2014;121(5):990–8. doi: 10.1097/ALN.0000000000000436 . 25233374 10.1097/ALN.0000000000000436 PMC4206606

[15] Vlisides PE, Bel-Bahar T, Lee U, Li D, Kim H, Janke E, et al Neurophysiologic Correlates of Ketamine Sedation and Anesthesia: A High-density Electroencephalography Study in Healthy Volunteers. Anesthesiology. 2017;127(1):58–69. doi: 10.1097/ALN.0000000000001671 . 28486269 10.1097/ALN.0000000000001671 PMC5478453

[16] Akeju O, Pavone KJ, Westover MB, Vazquez R, Prerau MJ, Harrell PG, et al A comparison of propofol- and dexmedetomidine-induced electroencephalogram dynamics using spectral and coherence analysis. Anesthesiology. 2014;121(5):978–89. doi: 10.1097/ALN.0000000000000419 . 25187999 10.1097/ALN.0000000000000419 PMC4304638

[17] Huupponen E, Maksimow A, Lapinlampi P, Sarkela M, Saastamoinen A, Snapir A, et al Electroencephalogram spindle activity during dexmedetomidine sedation and physiological sleep. Acta Anaesthesiol Scand. 2008;52(2):289–94. Epub 2007/11/17. doi: 10.1111/j.1399-6576.2007.01537.x . 18005372 10.1111/j.1399-6576.2007.01537.x

[18] Kasuya Y, Govinda R, Rauch S, Mascha EJ, Sessler DI, Turan A. The correlation between bispectral index and observational sedation scale in volunteers sedated with dexmedetomidine and propofol. Anesth Analg. 2009;109(6):1811–5. Epub 2009/11/20. doi: 10.1213/ANE.0b013e3181c04e58 . 19923507 10.1213/ANE.0b013e3181c04e58

[19] Schnider TW, Minto CF, Gambus PL, Andresen C, Goodale DB, Shafer SL, et al The influence of method of administration and covariates on the pharmacokinetics of propofol in adult volunteers. Anesthesiology. 1998;88(5):1170–82. . 9605675 10.1097/00000542-199805000-00006

[20] Zhang M, Zhang G, Yang J, Chen AC. The impact of a regular blood donation on the hematology and EEG of healthy young male blood donors. Brain topography. 2012;25(1):116–23. Epub 2011/10/14. doi: 10.1007/s10548-011-0203-0 . 21993841 10.1007/s10548-011-0203-0

[21] Delorme A, Makeig S. EEGLAB: an open source toolbox for analysis of single-trial EEG dynamics including independent component analysis. J Neurosci Methods. 2004;134(1):9–21. doi: 10.1016/j.jneumeth.2003.10.009 . 15102499 10.1016/j.jneumeth.2003.10.009

[22] Blain-Moraes S, Tarnal V, Vanini G, Alexander A, Rosen D, Shortal B, et al Neurophysiological correlates of sevoflurane-induced unconsciousness. Anesthesiology. 2015;122(2):307–16. doi: 10.1097/ALN.0000000000000482 ; PubMed Central PMCID: PMCPMC4301983. 25296108 10.1097/ALN.0000000000000482 PMC4301983

[23] Constant I, Sabourdin N. The EEG signal: a window on the cortical brain activity. Paediatr Anaesth. 2012;22(6):539–52. Epub 2012/05/19. doi: 10.1111/j.1460-9592.2012.03883.x . 22594406 10.1111/j.1460-9592.2012.03883.x

[24] Rampil IJ. A primer for EEG signal processing in anesthesia. Anesthesiology. 1998;89(4):980–1002. . 9778016 10.1097/00000542-199810000-00023

[25] Kuyrukluy?ld?z U, Binici O, Onk D, Ayhan Celik S, Torun MT, Unver E, et al Comparison of dexmedetomidine and propofol used for drug-induced sleep endoscopy in patients with obstructive sleep apnea syndrome. Int J Clin Exp Med. 2015;8(4):5691–8. . 26131153 PMC4483890

[26] Ngwenyama NE, Anderson J, Hoernschemeyer DG, Tobias JD. Effects of dexmedetomidine on propofol and remifentanil infusion rates during total intravenous anesthesia for spine surgery in adolescents. Paediatr Anaesth. 2008;18(12):1190–5. doi: 10.1111/j.1460-9592.2008.02787.x . 19076573 10.1111/j.1460-9592.2008.02787.x

[27] Wang T, Ge S, Xiong W, Zhou P, Cang J, Xue Z. Effects of different loading doses of dexmedetomidine on bispectral index under stepwise propofol target-controlled infusion. Pharmacology. 2013;91(1–2):1–6. doi: 10.1159/000343634 . 23095710 10.1159/000343634

[28] Le Guen M, Liu N, Tounou F, Auge M, Tuil O, Chazot T, et al Dexmedetomidine reduces propofol and remifentanil requirements during bispectral index-guided closed-loop anesthesia: a double-blind, placebo-controlled trial. Anesth Analg. 2014;118(5):946–55. doi: 10.1213/ANE.0000000000000185 . 24722260 10.1213/ANE.0000000000000185

[29] Liu X, Lauer KK, Ward BD, Li SJ, Hudetz AG. Differential effects of deep sedation with propofol on the specific and nonspecific thalamocortical systems: a functional magnetic resonance imaging study. Anesthesiology. 2013;118(1):59–69. doi: 10.1097/ALN.0b013e318277a801 . 23221862 10.1097/ALN.0b013e318277a801 PMC4080838

[30] Brown EN, Purdon PL, Van Dort CJ. General anesthesia and altered states of arousal: a systems neuroscience analysis. Annu Rev Neurosci. 2011;34:601–28. doi: 10.1146/annurev-neuro-060909-153200 . 21513454 10.1146/annurev-neuro-060909-153200 PMC3390788

[31] Nelson LE, Guo TZ, Lu J, Saper CB, Franks NP, Maze M. The sedative component of anesthesia is mediated by GABA(A) receptors in an endogenous sleep pathway. Nat Neurosci. 2002;5(10):979–84. doi: 10.1038/nn913 . 12195434 10.1038/nn913

[32] Nelson LE, Lu J, Guo T, Saper CB, Franks NP, Maze M. The alpha2-adrenoceptor agonist dexmedetomidine converges on an endogenous sleep-promoting pathway to exert its sedative effects. Anesthesiology. 2003;98(2):428–36. Epub 2003/01/29. . 12552203 10.1097/00000542-200302000-00024

[33] Akeju O, Loggia ML, Catana C, Pavone KJ, Vazquez R, Rhee J, et al Disruption of thalamic functional connectivity is a neural correlate of dexmedetomidine-induced unconsciousness. Elife. 2014;3:e04499 Epub 2014/11/29. doi: 10.7554/eLife.04499 ; PubMed Central PMCID: PMCPMC4280551. 25432022 10.7554/eLife.04499 PMC4280551

[34] Flores FJ, Hartnack KE, Fath AB, Kim SE, Wilson MA, Brown EN, et al Thalamocortical synchronization during induction and emergence from propofol-induced unconsciousness. Proc Natl Acad Sci U S A. 2017;114(32):E6660–E8. doi: 10.1073/pnas.1700148114 . 28743752 10.1073/pnas.1700148114 PMC5558998

[35] Cantero JL, Atienza M, Salas RM. Human alpha oscillations in wakefulness, drowsiness period, and REM sleep: different electroencephalographic phenomena within the alpha band. Neurophysiol Clin. 2002;32(1):54–71. Epub 2002/03/28. . 11915486 10.1016/s0987-7053(01)00289-1

[36] Bhattacharya BS, Patterson C, Galluppi F, Durrant SJ, Furber S. Engineering a thalamo-cortico-thalamic circuit on SpiNNaker: a preliminary study toward modeling sleep and wakefulness. Front Neural Circuits. 2014;8:46 doi: 10.3389/fncir.2014.00046 . 24904294 10.3389/fncir.2014.00046 PMC4033042

[37] Cantero JL, Atienza M, Salas RM, Gomez CM. Alpha EEG coherence in different brain states: an electrophysiological index of the arousal level in human subjects. Neurosci Lett. 1999;271(3):167–70. Epub 1999/10/03. . 10507695 10.1016/s0304-3940(99)00565-0

[38] Andrillon T, Nir Y, Staba RJ, Ferrarelli F, Cirelli C, Tononi G, et al Sleep spindles in humans: insights from intracranial EEG and unit recordings. J Neurosci. 2011;31(49):17821–34. doi: 10.1523/JNEUROSCI.2604-11.2011 . 22159098 10.1523/JNEUROSCI.2604-11.2011 PMC3270580

[39] Baker R, Gent TC, Yang Q, Parker S, Vyssotski AL, Wisden W, et al Altered activity in the central medial thalamus precedes changes in the neocortex during transitions into both sleep and propofol anesthesia. J Neurosci. 2014;34(40):13326–35. doi: 10.1523/JNEUROSCI.1519-14.2014 . 25274812 10.1523/JNEUROSCI.1519-14.2014 PMC4180471

[40] Hill S, Tononi G. Modeling sleep and wakefulness in the thalamocortical system. J Neurophysiol. 2005;93(3):1671–98. Epub 2004/11/13. doi: 10.1152/jn.00915.2004 . 15537811 10.1152/jn.00915.2004

[41] Bollimunta A, Mo J, Schroeder CE, Ding M. Neuronal mechanisms and attentional modulation of corticothalamic alpha oscillations. J Neurosci. 2011;31(13):4935–43. doi: 10.1523/JNEUROSCI.5580-10.2011 . 21451032 10.1523/JNEUROSCI.5580-10.2011 PMC3505610

[42] Contreras D, Steriade M. Spindle oscillation in cats: the role of corticothalamic feedback in a thalamically generated rhythm. J Physiol. 1996;490 (Pt 1):159–79. . 8745285 10.1113/jphysiol.1996.sp021133 PMC1158654

[43] Vijayan S, Kopell NJ. Thalamic model of awake alpha oscillations and implications for stimulus processing. Proc Natl Acad Sci U S A. 2012;109(45):18553–8. doi: 10.1073/pnas.1215385109 . 23054840 10.1073/pnas.1215385109 PMC3494906

[44] Ching S, Brown EN. Modeling the dynamical effects of anesthesia on brain circuits. Curr Opin Neurobiol. 2014;25:116–22. doi: 10.1016/j.conb.2013.12.011 . 24457211 10.1016/j.conb.2013.12.011 PMC4181389

[45] Lee KH, McCormick DA. Abolition of spindle oscillations by serotonin and norepinephrine in the ferret lateral geniculate and perigeniculate nuclei in vitro. Neuron. 1996;17(2):309–21. Epub 1996/08/01. . 8780654 10.1016/s0896-6273(00)80162-2

[46] Ozgoren M, Bayazit O, Gokmen N, Oniz A. Spectral pattern analysis of propofol induced spindle oscillations in the presence of auditory stimulations. Open Neuroimag J. 2010;4:121–9. doi: 10.2174/1874440001004010121 . 21792383 10.2174/1874440001004010121 PMC3141347

[47] De Gennaro L, Ferrara M. Sleep spindles: an overview. Sleep Med Rev. 2003;7(5):423–40. Epub 2003/10/24. . 14573378 10.1053/smrv.2002.0252

[48] Molle M, Bergmann TO, Marshall L, Born J. Fast and slow spindles during the sleep slow oscillation: disparate coalescence and engagement in memory processing. Sleep. 2011;34(10):1411–21. doi: 10.5665/SLEEP.1290 ; PubMed Central PMCID: PMCPMC3174843. 21966073 10.5665/SLEEP.1290 PMC3174843

[49] Yordanova J, Kirov R, Verleger R, Kolev V. Dynamic coupling between slow waves and sleep spindles during slow wave sleep in humans is modulated by functional pre-sleep activation. Sci Rep. 2017;7(1):14496 Epub 2017/11/05. doi: 10.1038/s41598-017-15195-x . 29101344 10.1038/s41598-017-15195-x PMC5670140

[50] Piantoni G, Halgren E, Cash SS. The Contribution of Thalamocortical Core and Matrix Pathways to Sleep Spindles. Neural Plast. 2016;2016:3024342 doi: 10.1155/2016/3024342 . 27144033 10.1155/2016/3024342 PMC4842069

[51] Turan A, Dalton JE, Kasuya Y, Akca O, Sessler DI, Rauch S. Correlation between bispectral index, observational sedation scale, and lower esophageal sphincter pressure in volunteers using dexmedetomidine or propofol. Med Sci Monit. 2012;18(10):CR593–6. doi: 10.12659/MSM.883484 . 23018351 10.12659/MSM.883484 PMC3560568

[52] Park KS, Hur EJ, Han KW, Kil HY, Han TH. Bispectral index does not correlate with observer assessment of alertness and sedation scores during 0.5% bupivacaine epidural anesthesia with nitrous oxide sedation. Anesth Analg. 2006;103(2):385–9, table of contents. Epub 2006/07/25. doi: 10.1213/01.ane.0000226090.13170.25 . 16861421 10.1213/01.ane.0000226090.13170.25

[53] Isik B, Tuzuner T, Tezkirecioglu M, Oztas N. Nitrous oxide sedation and bispectral index. Eur J Dent. 2007;1(4):240–5. . 19212474 PMC2609910

[54] Nunes RR, Cavalcante SL, Franco SB. [Effects of sedation produced by the association of midazolam and ketamine s(+) on encephalographic variables]. Rev Bras Anestesiol. 2011;61(3):304–10. Epub 2011/05/21. doi: 10.1016/S0034-7094(11)70036-8 . 21596190 10.1016/S0034-7094(11)70036-8

[55] Suzuki M, Edmonds HL Jr., Tsueda K, Malkani AL, Roberts CS. Effect of ketamine on bispectral index and levels of sedation. J Clin Monit Comput. 1998;14(5):373 Epub 1999/02/10. . 9951765 10.1023/a:1009975701184

[56] Vereecke HE, Struys MM, Mortier EP. A comparison of bispectral index and ARX-derived auditory evoked potential index in measuring the clinical interaction between ketamine and propofol anaesthesia. Anaesthesia. 2003;58(10):957–61. Epub 2003/09/13. . 12969037 10.1046/j.1365-2044.2003.03403.x

[57] Hans P, Dewandre PY, Brichant JF, Bonhomme V. Comparative effects of ketamine on Bispectral Index and spectral entropy of the electroencephalogram under sevoflurane anaesthesia. Br J Anaesth. 2005;94(3):336–40. Epub 2004/12/14. doi: 10.1093/bja/aei047 . 15591328 10.1093/bja/aei047

[58] Faraoni D, Salengros JC, Engelman E, Ickx B, Barvais L. Ketamine has no effect on bispectral index during stable propofol-remifentanil anaesthesia. Br J Anaesth. 2009;102(3):336–9. Epub 2009/02/05. doi: 10.1093/bja/aen403 . 19189986 10.1093/bja/aen403

